# SD-OCT-histopathologic correlation in Schnabel’s cavernous optic nerve atrophy

**DOI:** 10.1038/s41433-025-03603-w

**Published:** 2025-01-18

**Authors:** C. Weber, K. Mercieca, J. M. Weller, L. M. Bulirsch, T. Ach, F. G. Holz, K. U. Loeffler, M. C. Herwig-Carl

**Affiliations:** 1 Department of Ophthalmology, University Hospital Bonn, Bonn, Germany; 2 Department of Ophthalmology, Friedrich-Alexander University Erlangen-Nürnberg, Erlangen, Germany; 3 CIO (Center for Integrated Oncology), University Hospital Bonn, Bonn, Germany; 4 Division of Ophthalmic Pathology, Department of Ophthalmology, University Hospital Bonn, Bonn, Germany

**Keywords:** Predictive markers, Nervous system

## Abstract

**Background:**

Until now, Schnabel’s cavernous optic nerve atrophy (SCONA) has solely been a histopathological diagnosis exhibiting variable degrees of optic nerve (ON) atrophy with characteristic cavernous spaces filled with acid mucopolysaccharides. We report the first correlation of histopathologic findings with spectral domain-optical coherence tomography (SD-OCT) imaging in SCONA.

**Methods:**

We examined the eye of an index patient with histopathologically identified SCONA who had undergone multimodal imaging before enucleation for iris ring melanoma. The extent of SCONA in the index patient and three other enucleated eyes with SCONA were determined histopathologically. The histopathological findings of our index patient were correlated with in vivo SD-OCT images before enucleation and compared to representative images from eyes with a normal versus glaucomatous optic disc.

**Results:**

Histopathologic examination of our index patient showed a pre- and intralaminar extension of SCONA. Atrophy of the inner retinal layers was observed corresponding to the extent of SCONA. Correlation with SD-OCT showed small intralaminar hyporeflective pseudocysts which were detected in multiple scans corresponding to the histologically affected areas. These changes were neither visible in scans of patients with glaucomatous atrophy nor those with a normal ON.

**Conclusions:**

We present the first correlation of clinical and pathological findings in SCONA and were able to identify distinct SD-OCT characteristics for this condition. These findings may help to detect SCONA in vivo and to study this rare entity clinically with regard to its clinical course, risk factors, and pathogenesis. However, more cases of SCONA are needed to confirm our findings.

## Introduction

Schnabel’s cavernous optic nerve atrophy (SCONA) was first described by Schnabel around 1900 [1–3]. Up to now, it has been a solely histopathological diagnosis showing characteristic cavernous spaces filled with acid mucopolysaccharides in parts of the optic nerve (ON), predominantly in the post-laminar region [4]. These cystoid spaces are characterized by accumulation of hyaluronic acid along with the loss of axons and myelin [5]. No signs of inflammation have been noted.

SCONA occurs unilaterally in most cases but can also be found bilaterally [4, 6–8]. It is often associated with a cupped or pale optic disc on clinical examination indicating some degree of ON atrophy [9–12]. Rarely, the ON was described clinically normal without signs of ON atrophy [7, 13].

The incidence of SCONA is unclear since it can only be detected histologically. Prevalence is estimated between 1.7% (mean age: 84 years in individuals with SCONA, w/o gender preponderance) [9] and 2.1% (mean age: 88 years in individuals with SCONA, 81% females) [4]. The aetiology of SCONA is still not completely understood. An association with cardiovascular diseases has been described in several case reports and in a case series by Giarelli et al. [7, 14, 15]. Some authors consider SCONA to be a variant of ischemic optic neuropathy [6, 16]. It can be found in eyes with glaucoma and has also been noted in eyes enucleated for uveal melanoma (UM) [9, 13, 14, 17].

SCONA can hitherto not be diagnosed by fundoscopy or with the aid of standard imaging tools. However, a clinical (in vivo) diagnosis using “more sophisticated high-resolution imaging” [4] could be beneficial to better understand the aetiology and pathogenesis of this disease. Currently, a variety of in vivo imaging modalities of the optic nerve head (ONH) are available. In particular, imaging by spectral-domain optic coherence tomography (SD-OCT) does not only allow for analysis of the nerve fiber layer but also for visualization of the ONH including parts of the lamina cribrosa.

Identification of patients with SCONA in vivo could not only help to study this rare disease but could also help with the differentiation of patients with SCONA from those with other optic neuropathies. Furthermore, regarding the possible association of SCONA with cardiovascular disease [10, 16], in vivo detection of SCONA may result in a subsequent medical screening to prevent patients from potential cardiovascular-related sequelae.

We present—to the best of our knowledge—the first correlation of histology and SD-OCT in an eye with SCONA. We propose that ON imaging by SD-OCT in SCONA presents characteristics different from glaucomatous ON atrophy or physiological findings and may help to diagnose this condition in vivo.

## Materials/subjects and methods

### Materials

**Fig. 1:**
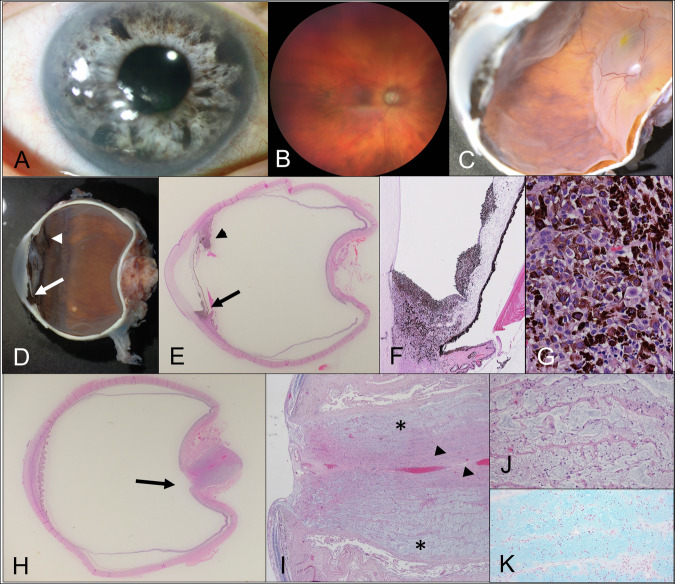
Index patient. **A** clinical picture OD showing a blue iris with many dark spots; **B** fundus image with optic nerve atrophy; **C** macroscopic image of the enucleated globe illustrating the posterior pole; **D** macroscopic image highlighting the iris ring melanoma (arrow) and the ciliary body lesion (arrow head); **E** corresponding histopathologic image [hematoxylin eosin (HE) stain, magnification 20×] with the iris ring melanoma (arrow) and the ciliary body lesion (arrow head); **F** higher magnification of the iris ring melanoma with infiltration of the inferior chamber angle, iris spots and vitreous seeds [HE stain, 100×]; **G** iris ring melanoma composed of pigmented and non-pigmented epithelioid cells [HE stain, 400×]; **H** histopathologic section showing the excavated optic nerve head (arrow) with Schnabel’s optic nerve atrophy (SCONA) [HE stain, 20×]; **I** optic nerve exhibiting SCONA (asterisk) and focal areas of normal optic nerve (arrow head) close to the central retinal vessels [HE stain, 40×]; **J** higher magnification of SCONA [HE stain, 400×]; **K** Alcian blue stain highlighting the acid mucopolysaccharides [Alcian blue, 400×].

We investigated one index patient with SCONA who had undergone multimodal imaging including SD-OCT with retinal nerve fiber layer (RNFL) and Bruch’s Membrane Opening (BMO) analysis (Heidelberg Engineering, Heidelberg, Germany) and fundus photography before enucleation for iris ring melanoma (Fig. 1). After enucleation, the eye was immediately fixed in 4% paraformaldehyde for 48 h and then sectioned vertically for best visualization of the tumor. Serial sections were made and the location of the sections was thoroughly documented.

Three additional cases of enucleated eyes with SCONA were analysed with regard to the extent of the histopathological findings. These eyes had been enucleated either for a blind, painful eye (*n* = 1), massive exposure keratitis (*n* = 1), or UM (*n* = 1) (Table 1). The diagnosis of SCONA was made after enucleation during histopathologic examination. SCONA was verified by Alcian blue staining in all four cases.

**Table. 1:** Demographic data and summary of the histopathologic findings of the four globes with SCONA [f female, m male, ON optic nerve, SCONA Schnabel’s cavernous optic nerve atrophy, yrs years].

Case	Demographic data	Clinical course	Volume of ON affected	Prelaminar extension of Schnabel’s atrophy	Maximum involvement of ON diameter	Further findings	Retina	ON excavation
1	f, 78 yrs	Secondary glaucoma due to iris ring melanoma	80%	Yes (and intralaminar)	100% (in some sections 60%, here predominantly superior)	Optic nerve atrophy	Global retinal atrophy (ganglion cell/nerve fiber layer/inner nuclear layer)	Severe
2	f, 42 yrs	Massive exposure keratitis in locked-in syndrome after intracerebral hemorrhage from an arterialvenous malformation	<50%	None (intralaminar)	60% (mostly nasally)	Cross section of ON w/o SCONA	Sectorial retinal atrophy corresponding to SCONA	Moderate
3	m, 71 yrs	Uveal melanoma (and glaucoma)	N/A	none	100%	Optic nerve atrophy	Global retinal atrophy (ganglion cell/nerve fiber layer)	Severe
4	m, 64 yrs	Vascularized corneal scar after recurrent herpetic keratitis, angle closure glaucoma, mature cataract, advanced glaucomatous optic atrophy	N/A	None (intralaminar)	80%	Optic nerve atrophy	Sectorial retinal atrophy (ganglion cell/nerve fiber layer) excluding the macula	Moderate

SD-OCT images of eyes with normal optic discs (*n* = 120) and eyes with significant glaucomatous optic neuropathy (GON, *n* = 92) were used for comparison.

The research was conducted in adherence to tenets of the Declaration of Helsinki. Ethics Board Approval from the Ethics Committee of the University of Bonn was granted (328/16).

### Methods

Our index case and three additional patients with SCONA were histopathologically graded on Hematoxylin-Eosin (H&E) stains for the ON involvement using a modified score introduced by Giarelli (grade I: ≤25%; grade II: 26–50%; grade III: 51–75%; grade IV: more than 75% of ON diameter involved by SCONA) [4]. Alcian blue stains for the detection of acid mucopolysaccharides were performed to verify the diagnosis of SCONA.

**Fig. 2:**
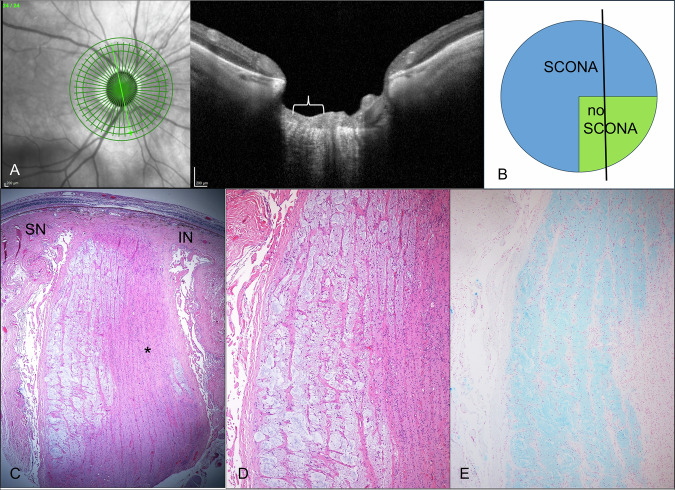
Correlation of SCONA. **A** Spectral domain-optic coherence tomography (SD-OCT) section through the optic nerve (ON). The left part (superotemporal, marked with parenthesis) of the ON shows septae of low reflectivity and cystoid spaces in a matrix of intermediate reflectivity. The right part of the optic nerve (inferonasal) shows a different morphology with hyperreflective septae. **B** Schematic picture of SCONA in our index patient based on the histological serial sections (blue: SCONA; green: area without SCONA). The lamina cribrosa is not affected in this section. This section does not correspond with the SD-OCT section. **C** Vertical histological section of the nasal part of the optic nerve head. SCONA is present in the superonasal (SN) part of the ON but mostly absent in the inferonasal (IN) part (asterisk; H&E stain, 40×). **D** Higher magnification of SCONA with amorphous material replacing the ON fibers (H&E stain, 100×). **E** Alcian blue stain highlighting mucopolysaccharides (100×).

**Fig. 3:**
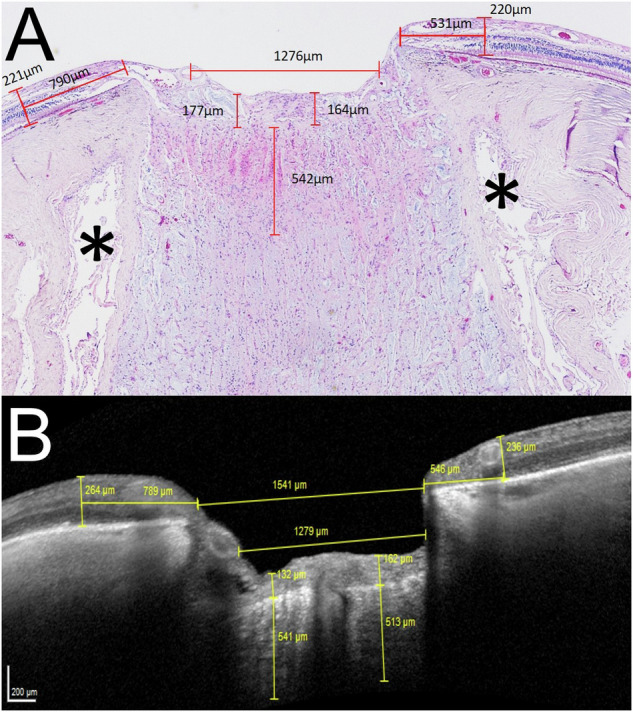
Measurements of histology and OCT. **A** Thickness measurements of the optic nerve head and adjacent retina in an H&E stained section of the index patient (central vertical section). The lamina cribrosa shows an extension of 542 µm. Optic nerve sheaths are marked by asterisk. **B** Thickness measurements of the optic nerve head and the adjacent retina in the corresponding vertical SD-OCT section (ONH mode, 1:1 pixel). It has to be acknowledged that histological tissues often undergo shrinkage during processing which may affect e.g., the retinal thickness in a way that the measurements in the histological sample can be slightly differ from in vivo OCT measurements.

SD-OCT images and H&E stained serial sections of the index patient were thoroughly correlated to identify corresponding structures (Fig. 2). For this, BMO-minimum rim width (MRW) modality with the ONH radial and circular (ONH-RC) scan, volume scans of the ON and RNFL scans were used in order to assess whether structures of SCONA were visible. The BMO-MRW modality with the ONH-RC scan was identified as best imaging mode to depict morphological changes of the ONH and thus, all parts of these scans were looked through and correlated with histopathological sections of different layers of the ON. Measurements of anatomical structures in the histopathological sections and the respective SD-OCT of the ONH-RC scans (Heidelberg Engineering measurement software) were also performed (Fig. 3). SD-OCT included acquisition of central 30° × 30° near-infrared reflectance (*λ* = S820 nm, automatic real time at least 15 frames) and had at least 19 B-Scans (distance between neighboring B-scans ≤240 µm). Further a volume scan of the ONH with at least 19 B-scans was performed as well. Overall, the BMO-MRW with the ONH-RC scan was used for comparison with other ONH since it had shown to be the best imaging modality to depict SCONA changes.

The structure and morphology of the ON in the SD-OCT images of our index case were compared with SD-OCT scans of eyes with GON (*n* = 92) and those with normal optic discs (*n* = 120).

## Results

### Index patient

The 78-year-old female index patient was referred to our tertiary referral center for persistent high intraocular pressure (IOP) and deteriorating visual acuity in her right eye (OD). Her OD best-corrected visual acuity was no light perception (NLP) and 20/20 on the Snellen chart in her left eye (OS). IOP at initial presentation was 38 mmHg OD under topical therapy with Brinzolamide/Brimonidine fixed-dose combination 3×/day; Timolol/Latanoprost fixed-dose combination at night, and 14 mmHg OS on no medication. In addition, the patient has a history of diabetes mellitus type 2 and systemic arterial hypertension. A nodular ulcerated melanoma had been excised from the left leg in 2019 and the patient was under treatment for carcinoma of the urinary bladder.

Slit-lamp examination showed a diffusely hyperpigmented iris OD (Fig. 1A). On gonioscopy, some of the brownish lesions had invaded the iridocorneal angle, in particular from three to nine o’ clock. There was an episcleral pigmentation at the six o’ clock position. The patient was pseudophakic in both eyes (OU). Fundoscopy OD showed an ONH atrophy with a cup-to-disc ratio of 0.95 and peripapillary atrophy, while the central and peripheral retina did not reveal any abnormalities (Fig. 1B, C). The OCT volume scans of the macula OD showed an atrophy of the inner retinal layers consistent with ON atrophy while the OS scans were unremarkable. In the right eye, RNFL thickness scan analysis demonstrated a clear circumferential thinning, especially in the superotemporal and inferotemporal quadrant. The posterior pole OCT scan showed a circular thinning of the ganglion cell layer, particularly in the temporal region. RNFL thickness scan analysis was unremarkable in OS.

Since the abovementioned findings were highly suspicious for iris ring melanoma, enucleation of OD was performed. Histopathological examination confirmed the diagnosis with a tumor composed mainly of epithelioid cells. There was diffuse infiltration of the iris and the chamber angle with focal extraocular growth as well as ciliary body involvement (classification according to the American Joint Committee on Cancer: T4a, G3, N0, M0). Furthermore, there was tumor seeding into the peripheral vitreous and along the lens capsule (Fig. 1D–G). Sonography of the neck and lymph nodes as well as the abdomen did not reveal any metastases. Genetic testing for a BAP1 germline mutation was negative. However, the tumor itself showed nuclear loss of the BAP1 immunoreaction highly indicative of a BAP1 mutation within the tumor. In addition, the ON of our patient showed SCONA with cavernous empty spaces occupying 80% of the ON (Fig. 1H–K).

### Characteristics of histopathological examinations of SCONA patients

The histopathological findings of our index case and three additional patients with SCONA revealed mucopolysaccharide deposits to a varying degree (Table 1). Except for our index case, no prelaminar extension of SCONA was detected in the control eyes showed intralaminar extension. All investigated eyes had either global or sectorial retinal atrophy corresponding to the degree of affected ON.

### SD-OCT-histopathological correlation (index patient)

**Fig. 4:**
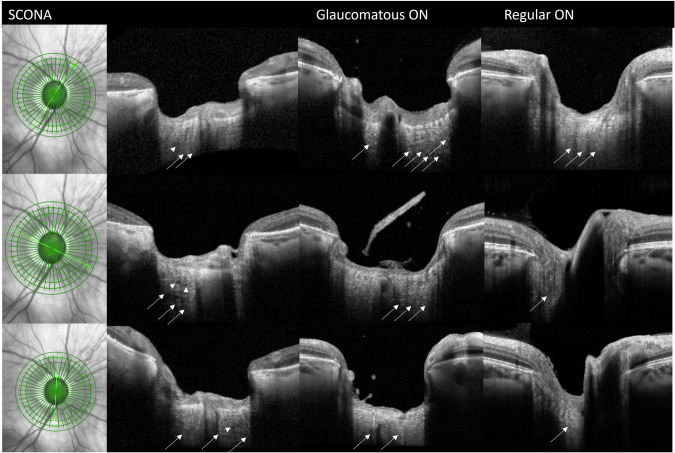
Bruch’s membrane opening minimum rim width with ONH radial and circular scans (BMO-MRW: ONH-RC). Imaging of our index patient with SCONA (Case 1) that had received both histological and OCT imaging showed cystoid spaces with minimal reflectivity (arrowheads) located within areas of medium reflectivity which represent areas of former nerve fibers as well as septae of predominantly low reflectivity (arrows). Exemplary cases of BMO-MRW with ONH-RC scan of patients with glaucomatous atrophy and regular ON are shown for comparison. No cystoid hyporeflective spaces were seen and the septae of glaucomatous eyes (arrows) were mainly hyperreflective. The scans of regular ON also displayed hyperreflective areas in the intralaminar region, but these changes did not have such a clear septate appearance (arrows). We further observed that the morphology of the optic nerve in regular ON was not as clearly visible as in glaucomatous ON.

The BMO-MRW modality with the ONH-RC scan showed small, round “pseudocysts” of nearly no reflectivity which were located within an area of medium reflectivity (representing the area of the former nerve fibers) could be detected in the intralaminar portion of the ON in several scans (Fig. 4). The presumed septae were also of low reflectivity in these particular areas where they usually show higher reflectivity. The pseudocysts surrounded by tissue of medium reflectivity correlated well with the parts of the ON that showed SCONA in our histopathologic analysis (Fig. 2).

SD-OCT measurements of different morphological structures (e.g., retinal thickness, width, and depth of ONH excavation) correlated well with the histologic sections at the corresponding anatomical region (Fig. 3). The ONH-RC scan was able to depict the ON head up to 541 µm depth which corresponds to the ONH including the lamina cribrosa. Thus, the ONH-RC only allow depiction of pre- and intra-laminar structures but does not enable an in vivo visualization of retrolaminar changes.

### Comparison of ONH-RC (our index patient) with OCT-scans from atrophic glaucomatous ON and regular ON

SC-OCT scans of normal eyes (*n* = 120) and eyes with complete GON atrophy defined as cup-to-disc ratio of 1.0 (*n* = 98) were analysed and the findings were compared to that of our index patient. Since SCONA is extremely rare, it is unlikely (but cannot be entirely excluded) that SCONA was present in the control cohorts. The ONH-RC scans of the patients with terminal glaucoma had a similar excavation as our index patient. However, the BMO-MRW of patients with regular ON was smaller compared to these eyes, as expected.

There was a variety of OCT morphology at the level of the lamina cribrosa: The ONH-RC scans from glaucomatous eyes showed more hyperreflective changes in the intralaminar region. In comparison, the septae of glaucomatous eyes were more often hyperreflective (which is in concordance with what can be expected from a histopathological perspective, see Supplementary Fig. 1). In a few scans of patients with glaucomatous eyes, small cystoid hyporeflective lesions were visible in the intralaminar portion of the scans whilst these were by far smaller than the cystoid lesions in our SCONA patients. Moreover, the intralaminar portion seemed less hyporeflective than in our index patient with SCONA. The scans of regular ON also displayed hyperreflective areas in the intralaminar region, but these changes did not have a septate appearance. We further observed that the morphology of the ON was not as clearly visible as in glaucomatous ON. We did not find any cystoid lesions or any cavernous lesions with nearly no reflectivity in these scans (Fig. 4). In addition, the SD-OCT morphologic changes of our SCONA patient were more “defined” and showed less “background noise” than the morphology of glaucomatous and normal ONH which might be attributed to the specific morphology of the ON in SCONA with loss of axons compensated by the accumulation of acid mucopolysaccharides but (in contrast to glaucoma) without thickening of the pial septae of the ON which may be seen in SD-OCT as hyperreflective strands.

## Discussion

This is—to the best of our knowledge—the first case of Schnabel’s cavernous optic atrophy (SCONA) with SD-OCT-histopathological correlation. Further, it is also the first case description of SCONA in an eye with iris ring melanoma. Since SCONA is relatively rare (with an incidence of around 2%), there has not yet been any report on the features of SCONA in multimodal imaging [4]. To date, the diagnosis of SCONA can only be made histopathologically. SCONA is most often associated with a clinically apparent ON atrophy. So far, it is impossible to differentiate a patient with SCONA from other optic neuropathies with clinical investigations.

The pathogenesis of SCONA has been under discussion for over 100 years and has not been clearly defined yet [4, 5, 16, 18]. An association with vascular diseases was proposed by Giarelli et al. [15]. Furthermore, Kanavi et al. proposed that the coincidence of glaucoma may increase the risk of SCONA in eyes with UM [9]. They analysed the association of glaucoma and UM in relation to SCONA and detected SCONA in 0.9% of eye enucleated for UM (*n* = 1985), in 1.3% in eyes enucleated for glaucoma (*n* = 155) and in 1.7% in eye bank eyes (*n* = 517). Their findings suggest that the occurrence of glaucoma may increase the risk of SCONA in eyes with UM [9]. However, they were not able to find an association between SCONA and UM. Since SCONA is more often found in eye bank eyes than in UM eyes, we believe that there is a bias since UM eyes are still relatively often enucleated followed by a thorough investigation. Other indications than UM for an enucleation are blind painful eyes (some of them already phthisical) or acute perforations/inflammatory processes. While in acute situations and phthisical eyes, SCONA is not expected, other painful blind eyes often undergo evisceration preventing the analysis of the ON and thus a diagnosis of potential SCONA being present.

Histopathologically, the extension of SCONA is variable. Giarelli et al. described that cavernous degenerations were often restricted to the retrolaminar part of the ON but were prelaminar in four (out of 93) cases (an intralaminar extension was not graded in their study)[4, 15]. However, in our study, three out of four SCONA eyes showed histopathological intralaminar changes with our index patient also showing prelaminar deposits. Other authors also reported cases with intra- or prelaminar mucopolysaccharide deposits [6, 19], all of them had a large extent of SCONA in common. This is important because we showed—in concordance with other studies [20]—that the maximum depth of the ONH-RC depicts only the intralaminar part of the ONH but not the retrolaminar portion. Kagemann et al. proposed an imaging protocol that enabled good visualization of the lamina cribrosa on OCT [21]. Inoue et al. evaluated 52 eyes of 30 patients with glaucoma or ocular hypertension showing that 3D SD-OCT images enable a precise imaging of the lamina cribrosa and its structures [22]. In summary, only SCONA with a large extent of intralaminar deposits may be detected by SD-OCT, and SCONA solely localized to the retrolaminar portion will be missed. However, Strouthidis et al. performed a study comparing ONH morphology by SD-OCT and serial histological sections in a normal monkey eye and concluded that (1) SD-OCT imaging of the ON accurately matches the histologic sections and (2) SD-OCT imaging only captures the anterior laminar surface [20]. This discrepancy to our findings with regard to depth of penetration may be explained by the fact that we evaluated a glaucomatous eye with significant ONH excavation with the loss of tissue allowing for better visualization of the lamina cribrosa. This explanation is somewhat in concordance with our findings in SD-OCT images of normal ON in which the structure of the lamina cribrosa was not as clearly visible as in glaucomatous eyes with a large excavation.

Enhanced-depth imaging OCT (EDI-OCT) allows better penetration through the retina and might offer an advantage regarding this problem [23]. Chien et al. study the assessment of lamina cribrosa structures via EDI-OCT and correlation with histology. They concluded that parts of retrolaminar columns identified on EDI-OCT matched those in histologic sections, suggesting that EDI-OCT might be able to depict retrolaminar structures partially [24]. However, their method was applied to dissected ex vivo eyes, so it still has to be shown that EDI-OCT can also visualize retrolaminar structures in vivo. Further, they did not conclude on the amount of retrolaminar extension an EDI-OCT was able to visualize. Another study on EDI-OCT and ON head drusen (ODD) concluded that EDI-OCT was an excellent technique with highest sensitivity near the inner sclera, that was able to visualize ODD above the lamina cribrosa, but not of the retrolaminar portion [25]. To the best of our knowledge, there is no study showing that EDI-OCT enables visualization of retrolaminar tissue in vivo. We had not performed EDI-OCT for our patient before enucleation since we were not aware that SCONA was present in her eye, this was discovered afterwards during histopathologic investigations, so we were not able to compare both OCT techniques.

Other lesions of the ON such as corpora amylacea of the ONH (which can also be present in different layers of the retina, [19]) may be visualized by SD-OCT in contrast to corpora arachnoideae (arachnoid bodies) which are located too posteriorly from the lamina cribrosa to be detected by SD-OCT (Supplementary Fig. 2). Pseudo-Schnabel’s cavernous degeneration which rarely develops secondary to intraocular silicon oil fill may also be visualized (Supplementary Fig. 2). [26, 27].

In our index patient, we found cystoid lesions of nearly no reflectivity in the intralaminar part of the ON in ONH-RC scan which correlated to the intralaminar area of SCONA. In order not to confound these cystoid lesions with other pathologies, we compared ONH-RC scans from 120 normal eyes and 92 eyes with significant glaucomatous atrophy. We noted a great variability amongst all ONH-RC scans. In order to ensure that our findings in the index patient were really associated with SCONA, we carefully evaluated all scans from normal (*n* = 120) and glaucomatous (*n* = 92) ON and were able to detect small cystic lesions of low reflectivity in the intralaminar region which were by far smaller than the cystoid lesions in our SCONA patient. It is known that pseudocysts are visible in the retina of patients with age-related macular degeneration and geographic atrophy or in patients with an atrophic ON [28, 29]. Further, all glaucomatous eyes showed hyperreflective changes within the intralaminar region compatible with thickened pial septae. As mentioned before, these septae were wider than in our SCONA patient and did not contain both regions of low and high reflectivity. Strouthidis et al. [20] also showed that the pial septae in a healthy monkey eye have a higher reflectivity compared to the nerve fibers. No cystoid lesions were present in any of the SD-OCT images of eyes with a normal ON. The genesis of glaucomatous cupping is complex. It is caused by thinning of the ON due to damage of retinal ganglion cells and atrophy of the nerve fiber layers due to increased IOP. Other causes, such as low ocular blood flow, low blood pressure, thinning of lamina cribrosa, etc. also play a role in the development of glaucomatous excavation [30, 31]. Peripapillary and macular retinoschisis have been proposed to be indicators of advanced glaucomatous cupping [32].

Our study shows that SCONA may be diagnosed in vivo using OCT imaging. This should be implemented in clinical practice in patients with non-glaucomatous optic neuropathy, especially in elderly women, and in uveal melanoma patients. The BMO-MRW with the ONH-RC scan appears to represent the most suitable imaging modality and should be used to detect SCONA in vivo. Diagnosing SCONA early can help to understand this condition and may optimize systemic treatment of these patients for cardiovascular disease. Some patients might be confounded with glaucoma and receive unnecessary surgery because of optic neuropathy progression. Further, cardiovascular risk factors should be monitored more closely in these patients in order to prevent potential cardiovascular-related sequelae, so an early diagnosis is essential for that.

There are some limitations to our study. The number of eyes included in this study is rather small. This is due to the fact that SCONA is a very rare disease and is rarely found in histopathologic investigations. The limited sample size of SCONA specimens did not allow to perform quantitative comparisons between SCONA, glaucomatous eyes, and eyes with normal ON head. It would be interesting to expand this study in the future, however it might take years to retrieve more cases. However, one could retrieve stronger and more robust data from further quantitative data regarding OCT measurements. Since we were only able to correlate OCT and histology findings for one patient (our index patient, case 1), the proposed imaging protocol of our group has to be reevaluated when performing studies with larger datasets so that consistency of imaging data can be checked. Furthermore, we were not able to perform a subgroup analysis in order to rule out confounding factors due to the small sample size. However, it would be useful to take into account possible confounding factors, such as cardiovascular risk factors (diabetes mellitus, arterial hypertension, etc.) that might have an influence on the ON in order to find out if systemic diseases have an influence on the disease. Furthermore, it has to be acknowledged that there might be discrepancies between histopathology and OCT measurements since histological tissue can undergo shrinkage during the processing which can slightly affect dimensions and measurements. We chose the ONH mode with 1:1 pixel for OCT in order to perform accurate measurements, the same is not entirely possible for histological sections. Further, we were only able to visualize the prelaminar part of the ON of our index patient with our imaging protocol including SD-OCT. An EDI-OCT had not been performed before enucleation, but it would be interesting to compare both techniques in future studies. Moreover, it would be interesting to perform longitudinal studies in the future to show via OCT imaging how SCONA develops after diagnosis to further understand this rare entity. Further, we did not perform a functional correlation since SCONA was discovered as a coincidental finding after enucleation in our included eyes. As of today, SCONA has been a solely histologic diagnosis and it was therefore impossible to correlate it with functional assessments. Most eyes that were diagnosed with SCONA had compromised functional parameters due to the underlying disease because of which the eye was enucleated. However, we have shown that SCONA can now be diagnosed using OCT imaging. This might enable further studies in the future in order to correlate function with structural findings and extent of SCONA. Combining function testing as part of in vivo studies with imaging of SCONA would provide further insights into the disease. It would also be interesting to expand the Giarelli score in order to incorporate clinical variables once more cases of SCONA will be diagnosed in vivo without other underlying diseases. In summary, due to the small sample size, it has to be mentioned that this study should be considered as a preliminary study. The findings from this study could help and guide future studies. However, one should not establish definite conclusions based on the small number of eyes included.

In conclusion, we were able to show that our index patient with SCONA differed from other glaucomatous and non-glaucomatous eyes with regard to the existence of hypo- and hyperreflective septae as well as the presence of cystoid lesions on OCT. These findings might help to detect SCONA clinically in in cases with intralaminar extension so that patients with SCONA could be differentiated from patients with other optic neuropathies including glaucomatous optic neuropathy. Moreover, these patients could be worked-up for cardiovascular diseases to prevent potential cardiovascular-related sequelae since SCONA is associated often with these diseases [4, 7, 8].

The BMO-MRW modality with the ONH-RC scan appears to represent a suitable imaging modality to detect SCONA in vivo allowing to study this entity clinically with regard to its clinical course, risk factors, and pathogenesis. However, further SD-OCT (in vivo or ex vivo)-histopathological correlations are needed to confirm or expand our results since at the moment we were only able to include preliminary data that has to be validated by larger studies in the future.

## Summary

### What was known before

- Schnabel’s cavernous optic nerve atrophy (SCONA) is a solely histopathologic diagnosis showing characteristic cavernous spaces filled with acid mucopolysaccharides in parts of the optic nerve (ON), predominantly in the post laminar region.
- The pathogenesis of SCONA is still unclear. However, an association with cardiovascular disease has been reported.

### What this study adds

- We present—to the best of our knowledge—the first correlation of histology and SD-OCT in a patient with SCONA who underwent enucleation for an iris ring melanoma.
- The BMO-minimum rim width (MRW) with the ONH radial and circular (ONH-RC) scan was identified as the best imaging mode to depict ONH morphologic changes.
- Small, round “pseudocysts” of nearly no reflectivity which were located within an area of medium reflectivity (representing the area of the former nerve fibers) were detected in the intralaminar portion of the ON in several scans corresponding to cavernous spaces of SCONA.
- Our findings might enable us to detect SCONA clinically in cases with intralaminar extension so that patients with SCONA can be differentiated from patients with other optic neuropathies. Furthermore, these patients could be screened for cardiovascular diseases to prevent potential cardiovascular-related sequelae possibly associated with SCONA.

## Supplementary information

Supplementary Figure 1

Supplementary Figure 2

eye-reporting-checklist

Supplementary material

## Data Availability

The data that support the findings of this study are not openly available due to reasons of sensitivity and are available from the corresponding author upon reasonable request. Data are located in controlled access data storage at University Eye Hospital of Bonn.
