# Supplementary figures and images for: SD-OCT-histopathologic correlation in Schnabel’s cavernous optic nerve atrophy
Source: Eye (Lond). 2025 Jan 18;39(6):1203–10. doi: 10.1038/s41433-025-03603-w (PMC11978856; doi:10.1038/s41433-025-03603-w)

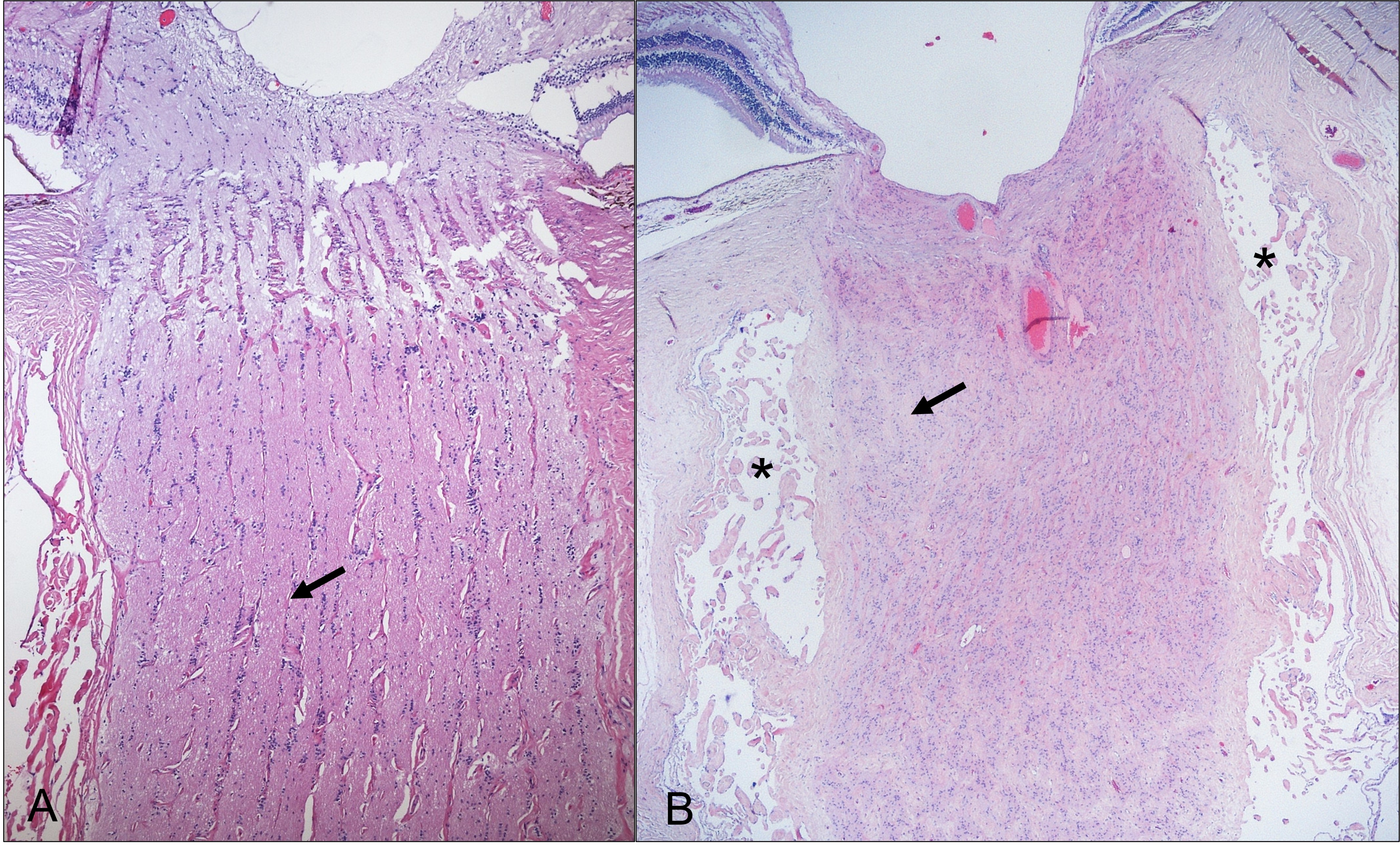

Supplement: Supplementary file 1 — Supplementary Figure 1 [file 41433_2025_3603_MOESM1_ESM.jpg]

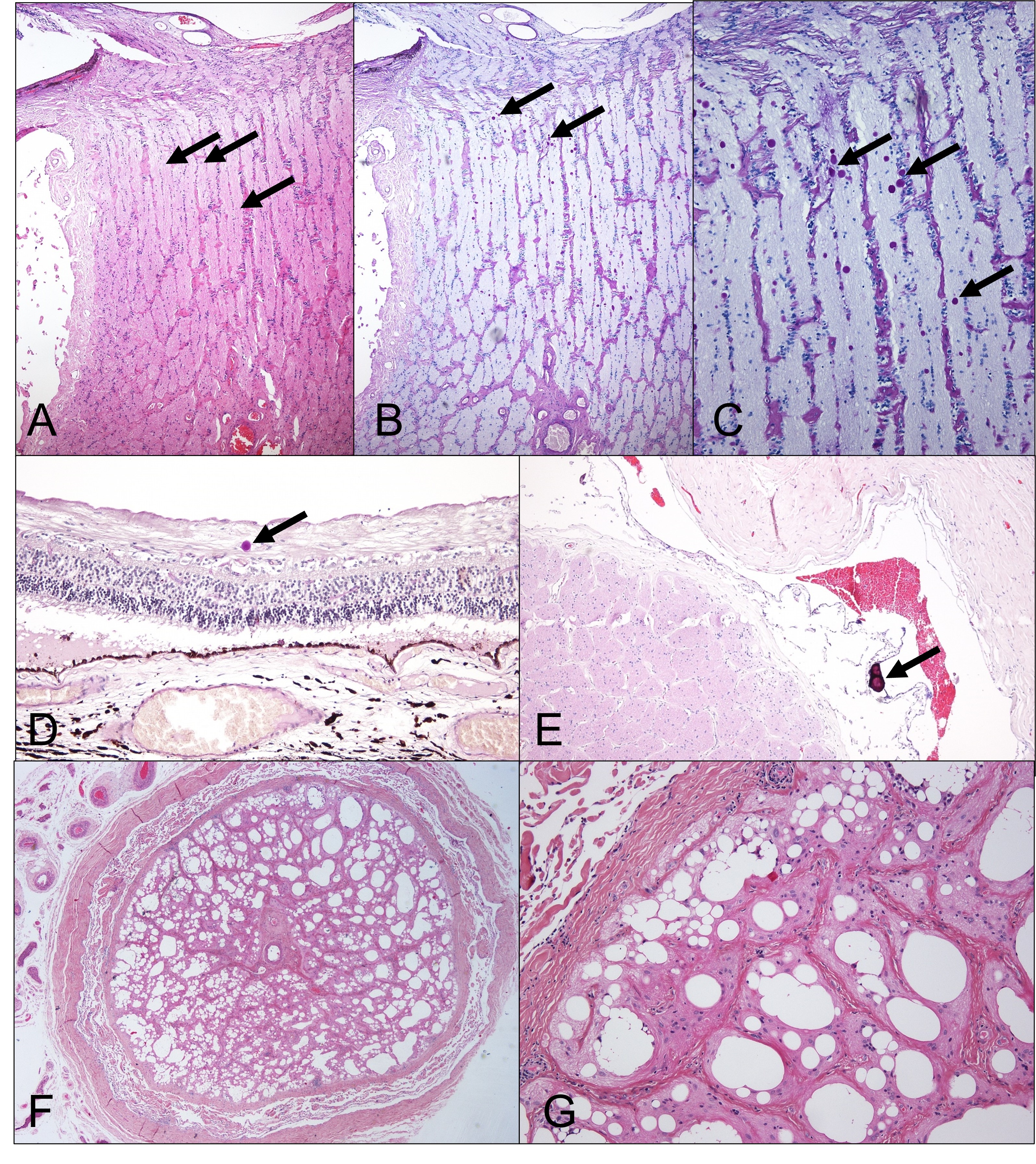

Supplement: Supplementary file 2 — Supplementary Figure 2 [file 41433_2025_3603_MOESM2_ESM.jpg]
