# Supplementary material for: SD-OCT-histopathologic correlation in Schnabel’s cavernous optic nerve atrophy
Source: Eye (Lond). 2025 Jan 18;39(6):1203–10. doi: 10.1038/s41433-025-03603-w (PMC11978856; doi:10.1038/s41433-025-03603-w)
Supplement: Supplementary file 4 — Supplementary material [file 41433_2025_3603_MOESM4_ESM.rtf]

Supplemental material
Supplemental Figure 1: 
Optic nerve histology. (A) Normal optic nerve with pial septae (arrow) surrounding the nerve fibers (H&E stain, 20x). (B)Glaucomatous optic nerve with. severe loss of nerve fibers and thickened fibrovascular pial septae (arrow). The nerve sheaths are "empty" due to ON shrinkage (asterisk; H&E stain, 20x) and an excavation of the ONHcan be also seen histologically. 

Supplemental Figure 2: 
Changes in the optic nerve and retina and their potential visualization on SD-OCT. (A) Corpora amylacea (arrows) in the optic nerve also affecting the lamina cribosa (H&E stain, 20x). (B) The amorphous round globules (arrows) are better visible with a PAS reaction (PAS, 20x). (C) Higher magnification shows clearly that the corpora amylacea (arrows) are present in the lamina cribrosa and retrolaminar (PAS, 40x). (D) Corpora amylacea (arrow) can be also found in the nerve fiber layer of the retina (PAS, 100x). (E) Corpora arachnoidea (arachnoid bodies, arrow) are found in the ON sheaths and will be too far posterior for SD-OCT imaging as shown in a cross section of the ON (H&E stain, 100x). (F) Pseudo Schnabel's atrophy with empty round spaces in the optic nerve due to silicon oil (H&E stain, 40x). (E) Higher magnification of F (H&E stain, 100x).
